# Autodetection of J Wave Based on Random Forest with Synchrosqueezed Wavelet Transform

**DOI:** 10.1155/2018/1315357

**Published:** 2018-07-03

**Authors:** Dengao Li, Xinyan Liu, Jumin Zhao, Jie Zhou

**Affiliations:** College of Information and Computer, Taiyuan University of Technology, Taiyuan, China

## Abstract

J wave is the bulge generated in the descending slope of the terminal portion of the QRS complex in the electrocardiogram. The presence of J wave may lead to sudden death. However, the diagnosis of J wave variation only depends on doctor's clinical experiences at present and missed diagnosis is easy to occur. In this paper, a new method is proposed to realize the automatic detection of J wave. First, the synchrosqueezed wavelet transform is used to obtain the precise time-frequency information of the ECG. Then, the inverse transformation of SST is computed to get the intrinsic mode function of the ECG. At last, the time-frequency features and SST-based and the entropy features based on modes are fed to Random forest to realize the automatic detection of J wave. As the experimental results shown, the proposed method has achieved the highest accuracy, sensitivity, and specificity compared with existing techniques.

## 1. Introduction

J wave is the bulge or ectrosis generated in the descending slope of the terminal portion of the QRS complex in the electrocardiogram (ECG). The morphologic pattern, amplitude, and the duration of J wave are various; besides, it always hides in the ST segment [1, 2]. The presence of J wave may lead to fatal malignant arrhythmia and even sudden death. Therefore, more and more attention has been attached to the research of J wave.

In 1936, Shipley and Hallaran discovered J wave in the ECG of patients with premature repolarization syndrome for the first time [3]. In 1938 Tomashewski found J wave in a frozen male patient's ECG [4]. In 1980s, the phenomenon of sudden death during sleep in young healthy men occurs frequently in Southeast Asian countries [5]. From 1948 to 1982 in Philippines, Manila, 722 cases of sudden death in healthy youth were reported and J wave occurred in their ECG [6–8]. In 1984, Otto et al. reported three healthy young men who had ventricular fibrillation during sleep, whose heart structures were normal, but the ECG showed J wave [9]. In 1992, Brugada brothers reported 8 cases with sudden cardiac death and J wave was found in their ECGs [10]. In 1996, Professor Yan and Professor Antzelevitch published an article in Circulation to investigate the molecular and electrophysiological principles of J wave [11]. Since then, the study of J wave has attracted more and more attention of experts and scholars, but these studies mainly focus on the view of medical science, and so far, only the doctor's clinical experience, combined with the naked eye to identify J wave appearing in the diagnosis of J wave syndrome [12]. However, the clinical misdiagnosis and missed diagnosis are easy to occur if the disease is diagnosed only by doctor's clinical experiences; because the morphologic pattern, amplitude, and the duration of J wave are various, the resulting symptoms are also different. Therefore, the automatic detection of J wave forms the perspective of signal processing and machine learning is a significant task.

At present, there are very few people who do this work. To the best of our knowledge, in 2014, Clack et al. analyzed the ECG with the help of computer for the first time. They set up a breakpoint at the descending slope of the QRS wave. As a result, they achieved the sensitivity of 89.5%, the specificity of 94.5%, and the accuracy of 91.3% [13]. In 2015, Wang et al. used signal processing combined with functional analysis to recognize J wave automatically, which achieved the sensitivity of 88.45%, the specificity of 87.8%, and the accuracy of 89.6% [14]. However, the datasets are too small and the results are not universal. In our previous work, we have used the curve fitting and wavelet transform to extract ECG features. Combined with SVM classifier, the sensitivity of 93.21%, the specificity of 93.87%, and the accuracy of 92.58% have been achieved [15]. However, at that time, the amount of data is too small, and the result is not convincing. The other drawback of this method is that the computational efficiency is not high. Since the incidence of J wave syndrome patients is low, we have tried to build J wave database in paper [16] before we have not collected enough samples. In that system, we have achieved the average sensitivity of 91.32%, average specificity of 92.2%, and average accuracy of 93.35%. But the built database, after all, is not real data, so we reexplore the methods of J wave automatic detection and identification after we collected enough data.

Wavelet transform (WT) is a good time-frequency analysis method, while it is restricted to the Heisenberg time-frequency uncertainty principle [17]. To put it another way, it is impossible for WT to improve the time-frequency resolution at the same time; that means, a high time resolution means a lower frequency resolution and vice versa. The temporal resolution and frequency resolution vary with the wavelet scale in WT, and the time-frequency blurring occurs on the transformed time-scale plane. Empirical mode decomposition (EMD) is an effective tool for time-frequency analysis of signals, while there are a lot of problems such as sifting criterion, endpoint effect, and mode mixing in EMD [18]. Moreover, the EMD does not have a firm mathematical framework. In view of the above shortcomings, ID et al. proposed a new time-frequency transform method named synchrosqueezed wavelet transform (SST). It is a powerful tool for time-frequency analysis of ECG and the precise time-frequency information can be evaluated using SST [19].

**Figure 1:**
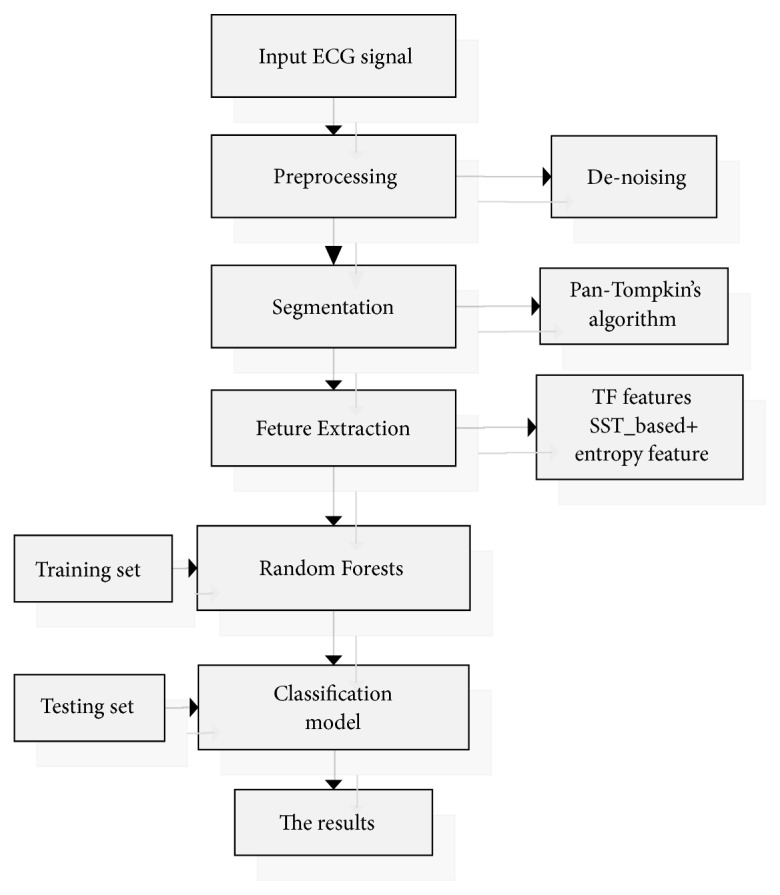
Block diagram of the proposed method.

In this paper, a new methodology based on SST and Random forest (RF) is proposed to realize the automatic detection of J wave. We computed the time-frequency feature based on SST as the first feature. Through the inverse transformation of SST we obtained five modes of the ECG episodes and we have evaluated Renyi entropy, approximate entropy, and sample entropy as the nonlinear features. Then, the RF is utilized to achieve the detection and classification of J wave-positive and J wave-negative from ECGs. The flow chart of the proposed method for the automatic detection of J wave is provided in Figure 1. The remaining part of the paper is organized as follows. In Section 2, we describe the database used in this work. In Section 3, the developed method is described. The results and discussion are presented in Section 4 and Section 5, respectively. Finally, the present work is concluded in Section 6.

## 2. Data Preparation

### 2.1. Data Source

In our work, the ECG signals were collected from the Shanxi Dayi Hospital, which is the cooperating partner of our project. Infiniti digital twelve-channel ECG SE_1200-Express was applied and the ECG data were sampled at 500 Hz. The database consisted of 30 normal ECG recordings (20 males and 10 females), which come from the health checkup, and 25 abnormal ECG recordings (23 males and 2 females), which come form the patients with J wave related diseases, and all human beings enrolled in the study were signed informed consent. We choose 20-minute duration of Holter monitoring for each ECG record. It is to say that we intercepted 1200 heart beats of each ECG recording in our research. In this paper, the normal ECG patter is defined as J wave-negative and the abnormal ECG patter is defined as J wave-positive. We divided the data into training sets and testing sets. Among them, the training sets contain 18 J wave-negative data and 15 J wave-positive data, while the testing sets are comprised of 12 J wave negative data and 10 J wave-positive data.

### 2.2. Preprocessing of Data

Denoising of the ECG signal is carried by eight level Daubechies wavelet 6 (db6) in this preprocessing stage [20]. Pan-Tompkin's algorithm is used for the detection of R-peak on the preprocessed ECG signal, after that the ECG episodes are segmented using the detected R-point [20, 21]. The number of the ECG beats for J wave-positive and J wave-negative used in this study are revealed in Table 1. Since J wave always hides in the ST segment, we choose 120 samples after R-point as our subjects in the study.

## 3. Method

### 3.1. Time-Frequency Feature SST-Based

#### 3.1.1. The Basic Theory of SST

SST is a powerful and promising tool to analyze the time-frequency (TF) information of nonstationary signals, which is based on WT and reallocation methods [22]. It is computed by reassigned wavelet coefficients from time-scale plane to TF-plane; thus, a sharper TF distribution is achieved. It is the postprocessing of WT. Besides, it succeeded the philosophy of the EMD. Different from EMD, it has a sound theoretical base and the mode mixing phenomenon has been overcome in SST. Another advantage of SST is that the kind of mother wavelet has a small part to play in the results of SST [19]. The basic principles of SST are as follows [23, 24].

The Continuous Wavelet Transform (CWT) of a signal is [25]

(1) $$\begin{array}{c} W_{f}\left(\;a,b\;\right)=\frac{1}{\sqrt{a}}\int f\left(\;t\;\right)\psi^{*}\left(\;\frac{t-b}{a}\;\right)dt \end{array}$$

where, *ψ* is the mother wavelet, *a* is the scaling factor, *b* is time shift factor.

According to Plancherel's theory, equation (1) can be rewritten as:

(2) $$\begin{array}{c} W_{f}\left(\;a,b\;\right)=\frac{1}{2\pi }{\int \hat{f}\left(\xi \right)a^{-1/2}\overset{\wedge }{\psi }}^{*}\left(\;a\xi \;\right)e^{jb\xi }d\xi \end{array}$$

where $\hat{\psi }(\xi )$ is the Fourier transform of *ψ*(*t*) and $\hat{f}(\xi )$is the Fourier transform of *f*(*t*).

When *f*(*t*) = *A* cos(*wt*), with Fourier transform $\hat{f}(\xi )=\pi A[\delta (\xi -w)+\delta (\xi +w)]$ then (2) can be transformed into

(3) $$\begin{array}{c} W_{f}\left(\;a,b\;\right)=\frac{A}{4\pi }a^{1/2}\bar{\overset{\wedge }{\psi }\left(aw\right)}e^{ibw} \end{array}$$

One of the properties of WT is that the TF energy of the results always concentrated around the central frequency of the signal. The most powerful place, commonly known as “ridge”, is the signal frequency. However, the energy smeared around the “ridge” always affects the recognition of the signal, which means, when $\hat{\psi }(\xi )$ is gathered around *ξ* = *w*_0_, *W*_*f*_(*a*, *b*) will be gathered around *a* = *w*_0_/*w*, while *W*_*f*_(*a*, *b*) will be diffused around the the “ridge” *a* = *w*_0_/*w*. On the other hand, the oscillation of *W*_*f*_(*a*, *b*) in *b* points to the original frequency *w*, nothing to do with the value of *a* [22]. This is the theoretical basis of SST.

The process of the SST is as follows [26, 27]:

(1) Calculate the frequency domain form of the results of WT, just as (3).
(2) Calculate the instantaneous frequency (IF) of the signal.
  (4) $$\begin{array}{c} w_{f}\left(\;a,b\;\right)=-i{\left(\;W_{f}\left(\;a,b\;\right)\;\right)}^{-1}\frac{\partial }{\partial b}W_{f}\left(a,b\right) \end{array}$$
(3) Discretize scaling factor *a* and compute the *W*_*f*_(*a*, *b*) for any *a*_*k*_.
(4) Compress and rearrange the coefficients of WT. The information can be transformed from the time-scale plane to the time-frequency plane; moreover, the IF can be extracted in this step.
  (5) Tfwl,b=Δw−1∑ak:wak,b−wl≤Δw/2Wfak,bak−3/2Δak

where Δ*w* = *w*_*l*_ − *w*_*l*−1_ and *a*_*k*_ − *a*_*k*−1_ = Δ*a*_*k*_.

Equation (5) reveals that the TF representation is obtained by “synchrosqueezing” along scale direction at the narrow band [*w*_*l*_ − Δ*w*/2, *w*_*l*_ + Δ*w*/2] with central frequency *w*_*l*_, i.e., the scaling factor *a* and the IF *w*_*f*_(*a*, *b*) are “binned” once the equation is computed.

#### 3.1.2. The Parameter Selection of SST

**Figure 2:**
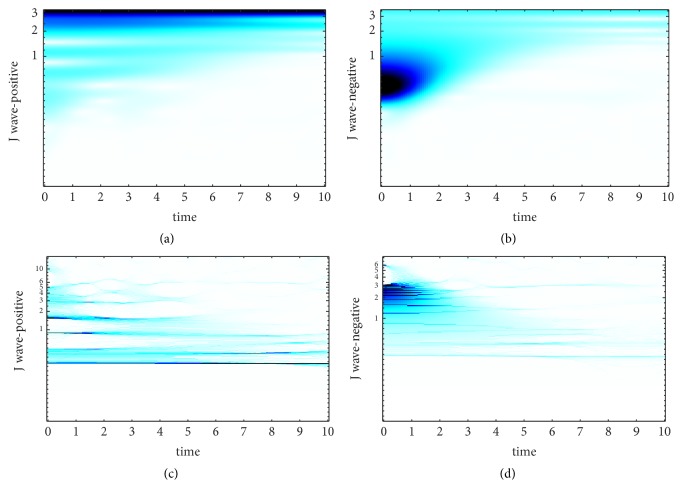
The time-frequency curve of the ECG fragment when the center frequency is 25hz. (a) The TF-plane WT-based of J wave-positive. (b) The TF-plane WT-based of J wave-negative. (c) The TF-plane SST-based of J wave-positive. (d) The TF-plane SST-based of J wave-negative.

**Figure 3:**
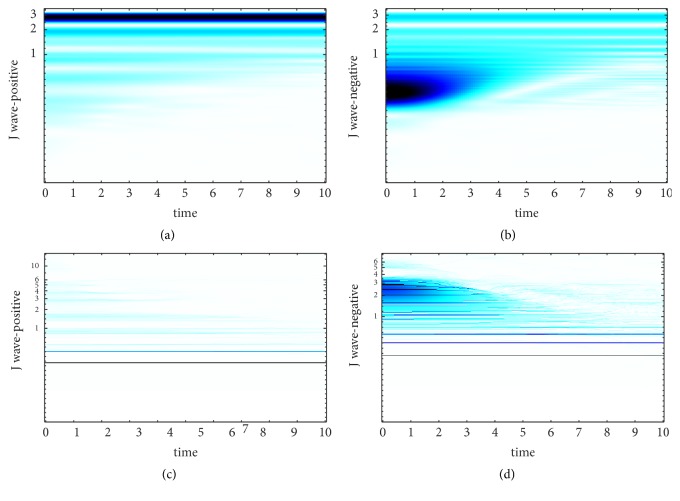
The time-frequency curve of the ECG fragment when the center frequency is 35hz. (a) The TF-plane WT-based of J wave-positive. (b) The TF-plane WT-based of J wave-negative. (c) The TF-plane SST-based of J wave-positive. (d) The TF-plane SST-based of J wave-negative.

**Figure 4:**
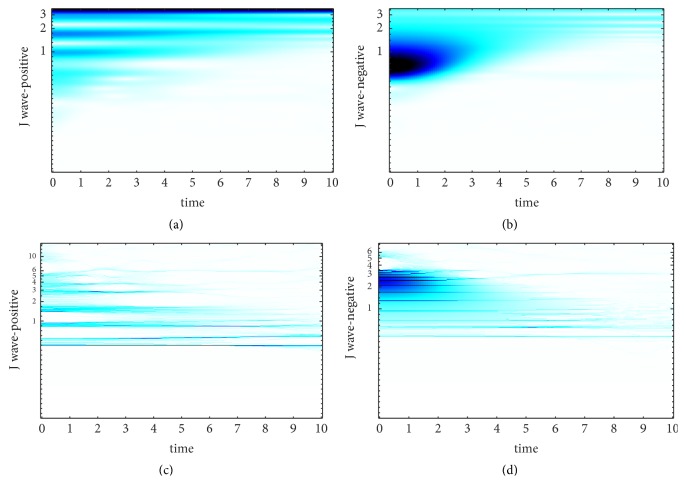
The time-frequency curve of the ECG fragment when the center frequency is 45hz. (a) The TF-plane WT-based of J wave-positive. (b) The TF-plane WT-based of J wave-negative. (c) The TF-plane SST-based of J wave-positive. (d) The TF-plane SST-based of J wave-negative.

SST is an improvement based on CWT. The choice of wavelet basis and the setting of wavelet base parameter make great differences to the results of CWT. In [25], It is proved that the wavelet base has much more smaller effect on SST compared with CWT and it is another advantage of SST. Morlet wavelet is carried in this paper, and we set the center frequencies of the wavelet basis are 25hz, 35hz, and 45hz, respectively to find the best center frequency of Morlet wavelet. The TF curve of J wave-positive and J wave-negative signal obtained by WT and SST at different center frequencies is revealed in Figures 2–4.

The TF-plane WT-based is obtained by (4). It can be shown from Figures 2–4 that the TF-plane derived from the WT is subjected to a poor TF resolution and smearing effect along frequency axis is serious. In contrast, the TF resolution SST-based is more focused and more energy-intensive. Besides, when the center frequency of wavelet basis is 35hz, we obtain the best TF resolution. Since the excellent performance of SST to achieve a high-precision TF resolution, we choose the results of SST as the first kind of feature to realize the automatic detection of J wave. SST can avoid frequency mixing effectively. Even the decomposed signal is contained of modes with relatively close frequency, SST can still extract them. This powerful function is based on the precise reconstruction theory of SST and the theories are as follows.

For any signal *f*(*t*), it can be indicated as *f*(*t*) = ∑_*k*=1_^*K*^*A*_*k*_(*t*)*e*^*iϕ*_*k*_(*t*)^ + *φ*(*t*) and the continuous form of *f*(*t*) SST-based is as follows:

(6) Sf,ε~δb,w=∫Aε,f~bWfa,b1δw−wfa,bδa−3/2da

where $\overset{\sim }{\varepsilon }$ is threshold and *δ* is accuracy and $A_{\overset{\sim }{\varepsilon },f(b)}=\{a\in R^{+};\left|W_{f}\left(a,b\right)\right|<\overset{\sim }{\varepsilon }\}$.

When $\overset{\sim }{\varepsilon }$ is small enough, it suffices to reconstruct each frequency mode signal with high precision [28]. That is, for each *k* = {1,2, 3....*K*}, constant *C* can be found, for any *b* ∈ *R*:

(7) limδ→0(Rψ−1∫w−ϕ′kb<ε~Sf,ε~δb,wdw−Akbejϕkb≤Cε~

where Rψ=2π∫ψ^(ζ)ζ-1dζ and *ϕ*′_*k*_(*b*) is the center frequency of every mode. The process to get the individual frequency modal function is as follows:

(1) Calculate the SST results of the original signal.

(2) Calculate the frequency center of individual component, that is, *ϕ*′_*k*_(*b*) [28].

(3) The discretized result of *T*_*f*_(*w*_*l*_, *b*) in (5) can be represented by ${\tilde{T}}_{\overset{\sim }{f}}(w_{l},t_{m})$, where *t*_*m*_ is the discrete time *t*_*m*_ = *t*_0_ + *m*Δ*t*, with Δ*t* being the sampling interval and *m* = 0,1, ...*n* − 1, n is the total number of modes contained in the signal *f*(*t*) [29].

(4) Calculate the individual component of *f*(*t*). It can be reconstructed from the ${\tilde{T}}_{\overset{\sim }{s}}(w_{l},t_{m})$ using the inverse CWT over a narrow frequency *τ* ∈ [*ϕ*′_*k*_ − (1/2)Δ*w*, *ϕ*′_*k*_ + (1/2)Δ*w*] around the *kth* component [30]. It can be evaluated as follows:

(8) $$\begin{array}{c} f_{k}\left(\;t_{m}\;\right)=2R_{\psi }^{-1}Re\left(\;\sum_{{\phi^{\prime }}_{k}\in \tau }{\tilde{T}}_{\overset{\sim }{S}}\left(\;{\phi^{\prime }}_{k},t_{m}\;\right)\;\right) \end{array}$$

**Figure 5:**
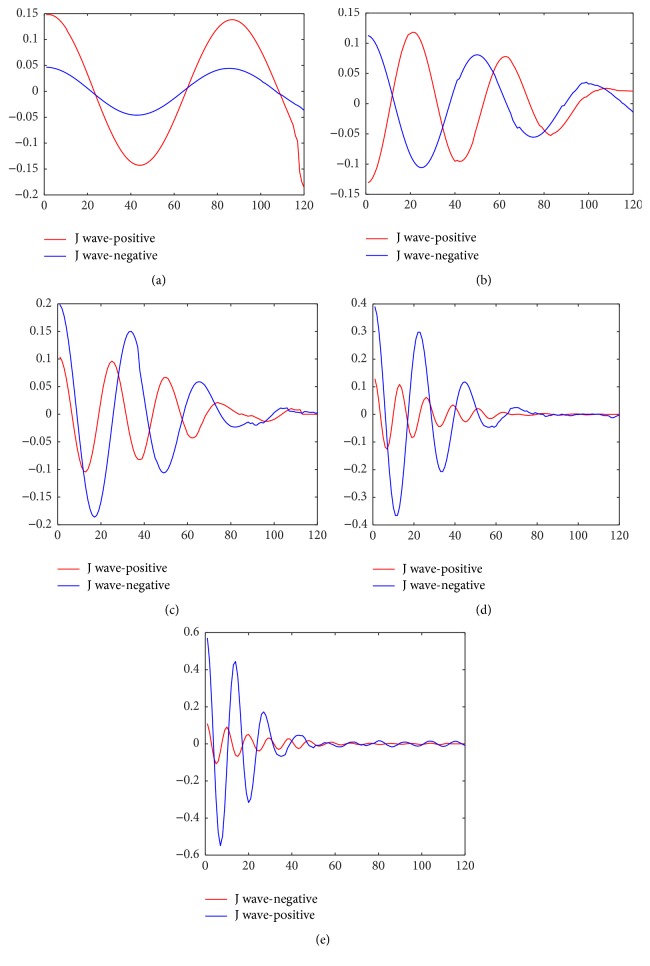
The intrinsic mode of J wave-positive and J wave-negative (a) mode 1, (b) mode 2, (c) mode 3, (d) mode 4, and (e) mode 5. The red lines stand for J wave-positive and the blue lines stand for J wave-negative.

Here, we get five modes and the intrinsic modes of J wave-positive and J wave-negative are provided in Figure 5.

It can be seen from Figure 5 that the amplitude and frequency information of J wave-positive and J wave-negative are distinguished significantly, especially at mode 3, mode 4, and mode 5. It is evident that the frequency characteristics of J wave-positive in mode 3, mode 4, and mode 5 are higher compared to the corresponding mode of J wave-negative, while the amplitude are lower.

The entropy features extracted in this paper are resulted from these intrinsic mode functions. It is discussed in the next subsection.

### 3.2. Nonlinear Entropy Feature Inverse SST-Based

Due to the nonlinear properties of biological signals, researchers tend to choose the theory of nonlinear dynamics, which are effective methods, to analyze them. When studying biological signals, entropy, as a kind of nonlinear feature, often makes a good performance. Renyi entropy (RE), approximate entropy (ApEn), and sample entropy (SampEn), for this reason, are used in this study to implement J wave automatic detection.

#### 3.2.1. Mode Renyi Entropy

In [31], Williams et al. introduced the RE of TF distribution. RE can be used as a measure of signal complexity at frequency domain, and the essence of signal can be researched by counting the RE of the signal at frequency domain [32]. Suppose that *X* is a random variable with a finite number of values. Its probability distribution is *p* = {*p*_1_, *p*_2_, ...*p*_*n*_} with *w*(*p*) = ∑*p*_*i*_ ≤ 1, and its RE is defined as

(9) $$\begin{array}{c} R^{\alpha }\left(\;p\;\right)=\frac{1}{1-\alpha }log_{2}\frac{\sum_{i}p_{i}^{\alpha }}{\sum_{i}p_{i}} \end{array}$$

When *α* = 1, the first-order RE degenerates into Shannon entropy. So, we regard RE as a more general form of information entropy. The theoretical derivation and simulation experiment in [33] concluded that when *α* = 3, the measurement of RE has the best stability. Therefore, the third-order RE can describe the information of different signals effectively. From what has been discussed above, we choose *α* = 3 in this article.

#### 3.2.2. Mode Approximate Entropy

ApEn is a kind of nonnegative quantitative description of the complexity of nonlinear time series. The more complex time series correspond to the greater value of ApEn [34]. Simultaneously, ApEn can obtain stable statistics even though the data is short. It is also for this reason, ApEn can achieve good performance in our work, which is defined as

(10) $$\begin{array}{c} Apen\left(\;m,r,N\;\right)=\frac{1}{N-m+1}\overset{N-m}{\underset{i=1}{\sum }}\log C_{i}^{m+1}\left(\;r\;\right) \end{array}$$

where *C*_*m*_^*i*^ is the correlation coefficient and it can be denoted as

(11) $$\begin{array}{c} C_{m}^{i}=\frac{1}{N-m+1}\overset{N-m+1}{\underset{j=1}{\sum }}\Theta \left(\;r-\left\|\;x_{i}-x_{j}\;\right\|\;\right) \end{array}$$

where *x*_*i*_, *x*_*j*_ represent phase trajectory points and *N*, *r*, Θ, *m* denote the number of midpoint in the phase space, radial length of a circular disk centered at the reference points, step function, and embedding dimension, respectively [34].

The performance of the ApEn is related to the values of *N*, *m*, and *r*. The results of the literature [34–36] show that when *m* is 2 and *r* is 0.2 multiple of the standard deviation of the data (SDNN), the value of ApEn has a steady statistical properties. Accordingly, we take *m* = 2 and *r* = 0.2 × *SDNN* in this work, respectively.

#### 3.2.3. Mode Sample Entropy

Proposed by Richman and Moornan, SampEn is similar to the ApEn but with higher precision to measure the complexity of the time series. For the sake of the value of SampEn, continuous matching of points inside the threshold *r* is done until the match does not exist [34, 37]. The variables *A*(*k*) and *B*(*k*) for all lengths *k* up to *e* keep track of all matching templates. It is given by

(12) $$\begin{array}{c} SampEn\left(\;k,r,N\;\right)=-\ln\frac{A\left(k\right)}{B\left(k-1\right)} \end{array}$$

where *k* = 0,1,…, *m* − 1 and *N* is the length of the study object. Similar to ApEn *m* = 2, *r* = 0.2 × *SDNN* are taken in this paper, respectively [37].

### 3.3. Classification

Combining with his Bagging Integrated Learning Theory proposed in 1996 and the random subspace method proposed by Ho in 1998, Leo Breiman introduced Random forest (RF) in 2001. RF is always regard as an excellent ensemble classifier [38]. The core of RF is to establish many decision trees according to random features from random samples with bagging strategy, and the final classification result is voted by these trees [39]. The classification processes of RF are given as follows:

(1) Adopt the technique of bootstrap resampling to extract multiple samples from the original samples.

(2) Build CART decision tree by selecting *K* features randomly from all features of above samples.

(3) Repeat the upper two steps *m* times, which is to set up the *m* CART decision trees.

(4) Combine multiple decision trees' prediction and draw a final classification results by voting.

RF is selected as the classifier to realize J wave automatic detection in this paper, since it has the following excellent properties compared to other classifiers:

(1) RF can deal with high-dimensional data and weak relevant data effectively [39–41].

(2) There is no overfitting in RF [40].

(3) It can draw the rank of importance of the features [40].

(4) There are less parameters which need to set in RF compared to other state-of-the-art classifiers. The number of the base decision trees is always the only variable need to set in RF; according to the study in [42, 43], the number of the base learners is set to 150 in this work.

## 4. Results

### 4.1. Analysis of Mode Entropy Features

**Figure 6:**
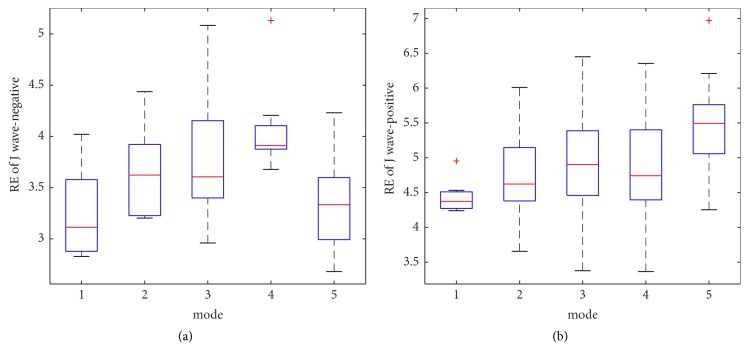
(a) The within-class variation of RE feature for J wave-negative from mode 1 to mode 5. (b) The within-class variation of RE feature for J wave-positive from mode 1 to mode 5.

**Figure 7:**
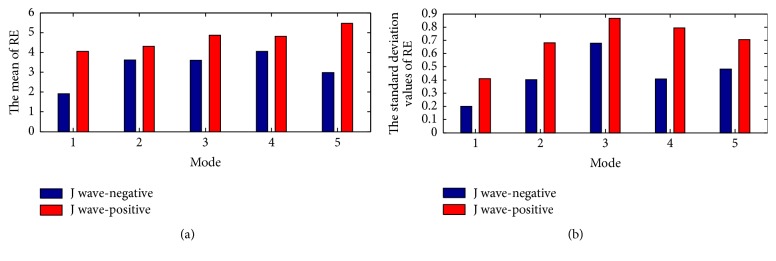
(a) The mean of RE from mode 1 to mode 5; (b) the standard deviation values of RE from mode 1 to mode 5.

In this subsection, we have analyzed the entropy features from the statistical perspective. The within-class variation of RE feature for J wave-negative and J wave-positive class from mode 1 to mode 5 is shown in Figures 6(a) and 6(b). Figures 7(a) and 7(b) depict the mean and the standard deviation values of RE. The results reveal that the mean and the standard deviation values of the J wave-positive episodes are higher than J wave-negative. In the analyses of the statistical significance of these features from mode 1 to mode 5, we have used Welch's two-tailed t-test technique [44, 45] by means of SPSS statistical analysis software. By doing this, t-value and p-value can be obtained, which are typically used to quantify the idea of statistical significance. The t-value and the p-value of RE from mode 1 to mode 5 have been listed in Table 2. The high t-value and the low p-value show that the discrimination of RE between J wave-negative and J wave-positive subjects are significant.

**Figure 8:**
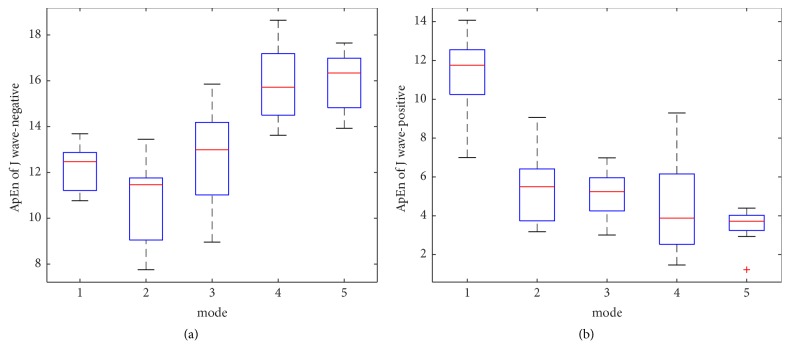
(a) The within-class variation of ApEn feature for J wave-negative from mode 1 to mode 5. (b) The within-class variation of ApEn feature for J wave-positive from mode 1 to mode 5.

**Figure 9:**
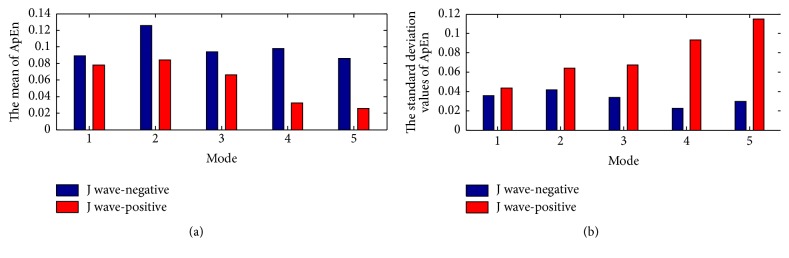
(a) The mean of ApEn from mode 1 to mode 5; (b) the standard deviation values of ApEn from mode 1 to mode 5.

**Figure 10:**
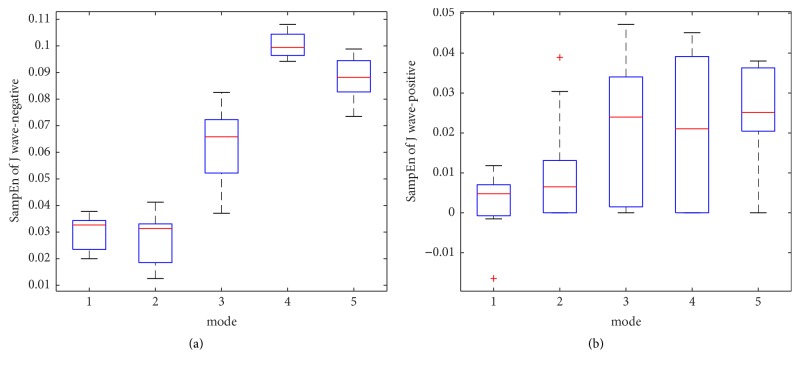
(a) The within-class variation of SampEn feature for J wave-negative from mode 1 to mode 5; (b) the within-class variation of SampEn feature for J wave-positive from mode 1 to mode 5.

**Figure 11:**
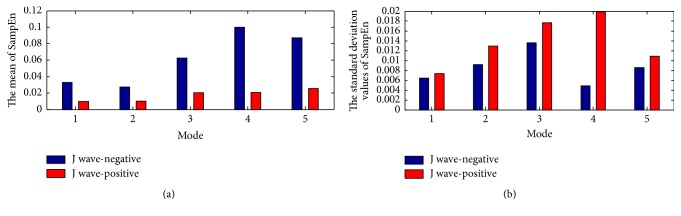
(a) The mean of SampEn from mode 1 to mode 5; (b) the standard deviation values of SampEn from mode 1 to mode 5.

The within-class variation of ApEn and SampEn feature for J wave-negative and J wave-positive class from mode 1 to mode 5 is shown in Figures 8(a), 8(b), 10(a), and 10(b), respectively. The mean and the standard deviation values of ApEn and SampEn are shown in Figures 9(a), 9(b), 11(a), and 11(b), respectively. From these figures, we have shown that the statistical features of ApEn and SampEn are significantly different for J wave-negative and J wave-positive episodes. The J wave-positive class has higher standard deviation values at mode 1 to mode 5, while inverse tendencies in the mean value of ApEn and SampEn from mode 1 to mode 5 are observed. The t-value and the p-value of ApEn and SampEn from mode 1 to mode 5 have been revealed in Tables 3 and 4. The p-values of the ApEn feature at mode 1, mode 2, and mode 5 are 0.003, 0.007, and 0.002, respectively. However, the p-values of the SampEn at mode 1 to mode 5 are less than 0.001; thus, the SampEn may have better performance in the process of the classification. Anyway, the entropy features are statistically significant for classification of J wave-negative and J wave-positive class from ECG and these features are suitable for detection of J wave-positive.

### 4.2. Performance Metrics

The following five types of performance evaluation indicators are used to evaluate the effect of the proposed method for ECG J wave detection [44, 46, 47]:

(13) SensitivitySe=TPTP+FN×100%SpecificitySp=TNTN+FP×100%AccuracyAcc=TP+TNTP+TN+FP+FN×100%Matthews  correlation  coefficientMCC=TP×TN−FP×FNTP+FPTN+FNTP+FNTN+FP

where true positive (TP) and false negative (FN) stand for the number of heartbeats of J-positive which have been classified correctly and incorrectly, respectively, while true negative (TN) and false positive (FP) stand for the number of heartbeats of J-negative which have been classified correctly and incorrectly, respectively. An ideal classification system should have lowered both FN and FP, so that it achieves high Se, high Sp, and high ACC as well as high MCC. In addition, the area under the receive operating characteristic curve (AUC) is used in our work to achieve more objective evaluation results and the higher the value of AUC, the more desirable the classification system.

### 4.3. Experimental Results

Firstly, we compared the presented method in this paper with some existing techniques and the results are listed in Table 5.

Table 5 revealed that the proposed method outperforms the methods reported in [13, 14]. The proposed method has achieved the highest ACC, Se, Sp, MCC, and AUC of 96.9%, 96.5%, 95.8%, 0.923, and 0.957, respectively. Besides, the databases of [13, 14] are too small. In [13] the database is comprised of 100 resting 12-lead samples. In [14] the training set contains 100 samples and the test set contains 116 samples, which results in the results of the experiment having no generality.

Secondly, the previous method we have reported in [15, 16] has been evaluated with the latest collected data and the results are shown in Table 6.

Table 6 revealed that the proposed method has achieved the highest ACC, Se, Sp, MCC, and AUC, which proved the effectiveness of the proposed method to realize the automatic detection of J wave.

## 5. Discussion

**Figure 12:**
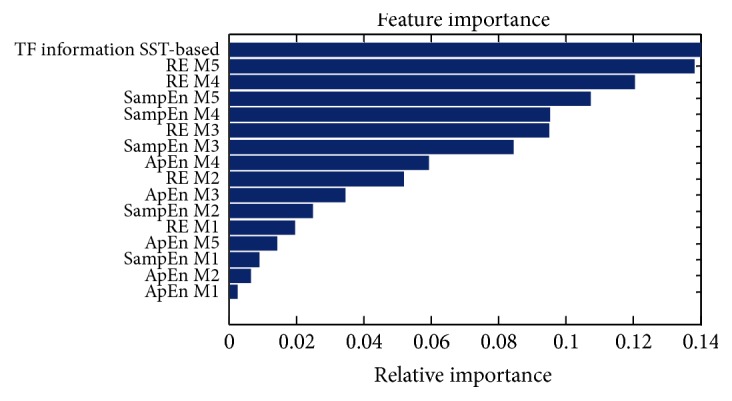
The ranking results of features extracted in this paper.

**Figure 13:**
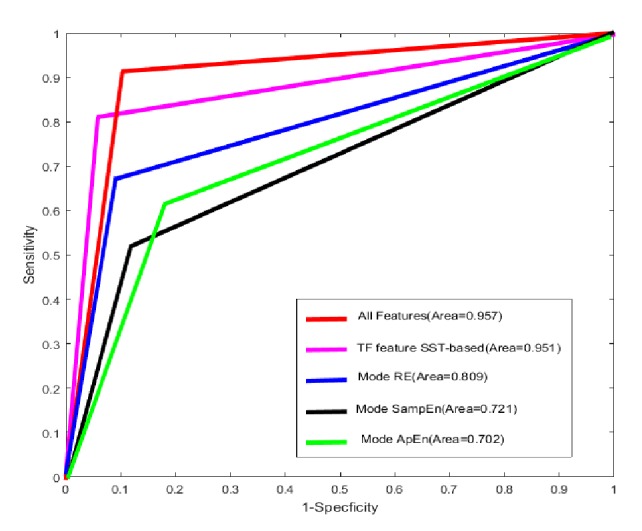
The ROC curve of RF classifier for various features.

In this subsection, the effect of different features on the classification results has been discussed firstly. RF can rank features according to the importance of the features; this is one of the superiorities of the RF.  Figure 12 depicts the ranking results. It is observed from Figure 12 that the time-frequency feature based on SST outperforms the other features. In the nonlinear entropy feature, the RE has the best performance and the effect of the SampEn is better than the ApEn. The RE feature emphasizes the spectral variation combined with the excellent time and frequency property of SST. This may be the reason for which the RE is ranked first in entropy feature. The received operating characteristic (ROC) curves of RF classifier for various features are provided in Figure 13. It can be seen that the RF classifier has the highest AUC when it is fed to all features (time-frequency feature, RE, ApEn, and SampEn feature) and the area is 0.957. The rest of area under ROC curves are 0.951, 0.809, 0.721, and 0.702 and the corresponding features are time-frequency feature, RE, ApEn, and SampEn feature, respectively, which is consistent with our earlier analysis.

In addition, the computational efficiency and detection results of different classifiers are discussed in this subsection.  Table 7 reveals the ACC, the value of MCC, AUC, the training time, and the testing time using the features extracted in this paper to different classifiers. For RF, the numbers of base learners are set as 100, 150, and 300, respectively. For K-Nearest Neighbour (KNN), the k is set as 6 [48]. For support vector machines (SVM), the idea of 10-fold cross validation and the grid-search is adopted to get the satisfactory parameter *γ* in radial basis function (RBF) and the penalty factor *C*, which had the shortest time-consuming [49, 50].

It can be seen from Table 7 that the RF is more time-consuming than KNN and DT in training sets, since it needs to establish many decision trees and votes for samples through the trees in the process of the training. It can also be observed that as the number of base learners increases, the training time and the testing time increase linearly. However, the testing time of RF is far less than its training time. In application, the testing time is more important, since the offline data is usually adopted in the process of testing. When the number of base learners is 150, the RF achieved the highest ACC, MCC, and AUC of 96.9%, 0.923, and 0.957, respectively, compared with other classifiers, which proved the sensible of choosing the classifier in our paper. Although SVM achieved comparable classification results to RF, its time consumption was much greater than that of RF.

## 6. Conclusion

A new method is proposed in this paper to achieve the automatic detection of J wave. The experimental results have proved that the proposed method can detect the J wave automatically and accurately. What is more, it provides a reliable foundation for the clinical diagnosis. We introduced time-frequency domain features and nonlinear entropy features (RE, ApEn, and SampEn) in the process of the feature extraction, after that, the RF is utilized in the stage of classification. The entropy features are computed by the modes of the ECG, which are evaluated by the inverse transformation of SST.

Compared with the existing techniques, the advantages of the proposed method are as follows. It is the first time to obtain the intrinsic mode function of ECG though SST. The good time-frequency characteristics and the perfect reconstruction ability of SST make it a powerful tool to discriminate J wave-negative and J wave-positive from ECGs. Combined with RF, which is a kind of ensemble classifiers with great performance, we obtain the best results to realize the automatic detection of J wave.

In the future, the work can be extended in two aspects:

(1) The developed methodology can be applied to the diagnosis and recognition of other heart diseases, even other biosignals, such as electroencephalogram(EEG).

(2) Feature selection can be studied to further improve the computational efficiency of J wave automatic detection.

## Figures and Tables

**Table 1:** The number of beats used in the work.

Set	J wave-negative	J wave-positive
Training set	21600	18000
Testing set	14400	12000
Total	36000	30000

**Table 2:** The t-value and the p-value of RE from mode 1 to mode 5.

Features	RE M1	RE M2	RE M3	RE M4	RE M5
t-value	20.82	19.95	18.64	19.89	17.96
p-value	<0.001	<0.001	<0.001	<0.001	<0.001

**Table 3:** The t-value and the p-value of ApEn from mode 1 to mode 5.

Features	ApEn M1	ApEn M2	ApEn M3	ApEn M4	ApEn M5
t-value	8.07	6.54	13.75	12.69	9.32
p-value	0.003	0.007	<0.001	<0.001	0.002

**Table 4:** The t-value and the p-value of SampEn from mode 1 to mode 5.

Features	SampEn M1	SampEn M2	SampEn M3	SampEn M4	SampEn M5
t-value	26.89	25.78	28.37	24.56	27.53
p-value	<0.001	<0.001	<0.001	<0.001	<0.001

**Table 5:** The results of existing techniques.

Method	ACC (%)	Se (%)	Sp (%)	MCC	AUC
Clark et al. [13]	91.3	89.5	94.5	0.840	0.893
Wang et al. [14]	89.6	88.5	87.8	0.760	0.852
The proposed method	96.9	96.5	95.8	0.923	0.957

**Table 6:** The results of the the previous method we have reported using the latest data.

Method	ACC (%)	Se (%)	Sp (%)	MCC	AUC
Li and Liu et al. [15]	92.6	93.2	93.9	0.870	0.928
Li and Bai et al. [16]	93.4	91.3	92.2	0.850	0.899
The proposed method	96.9	96.5	95.8	0.923	0.957

**Table 7:** The ACC, MCC, AUC, and training and testing time (in seconds) of different classifiers.

Classifier		ACC	MCC	AUC	Training time	Testing time	Total time
RF	75	90.9%	0.819	0.875	28.583	2.735	31.318
	150	96.9%	0.923	0.957	56.879	5.625	62.504
	300	94.1%	0.896	0.913	113.427	9.673	123.100
KNN (K=2)		87.63%	0.712	0.846	0.953	45.797	46.750
DT		85.6%	0.672	0.823	2.9041	0.052	2.993
SVM		95.0%	0.908	0.935	84.475	45.539	130.014

## Data Availability

All data used and analyzed during the current study are available from the corresponding author on reasonable request.
